# Supplementary material for: Measuring the orbital angular momentum spectrum of an electron beam
Source: Nat Commun. 2017 May 24;8:15536. doi: 10.1038/ncomms15536 (PMC5458084; doi:10.1038/ncomms15536)
Supplement: Supplementary Information — Supplementary Figures and Supplementary Notes. [file ncomms15536-s1.pdf]

Supplementary Figures

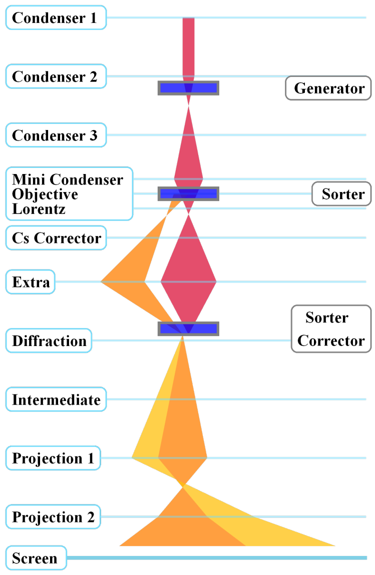

Supplementary Figure 1: **Experimental configuration of the electron sorter depicting the ray path of the electron beam.** Zeroth diffraction orders from our holograms are plotted in the same colour as that of the incident beam's while the first diffraction orders containing the hologram's phase information are plotted in different colours. Light blue lines depict the position of our electron microscope's lenses in our configuration and are labeled on the left side of the figure. Blue rectangles illustrate the phase elements of our sorter as reported in Fig. 1 and are labeled on the right side of the figure. Here, the vertical positions of the lenses are semi-quantitatively accurate and no form of horizontal scaling is considered.

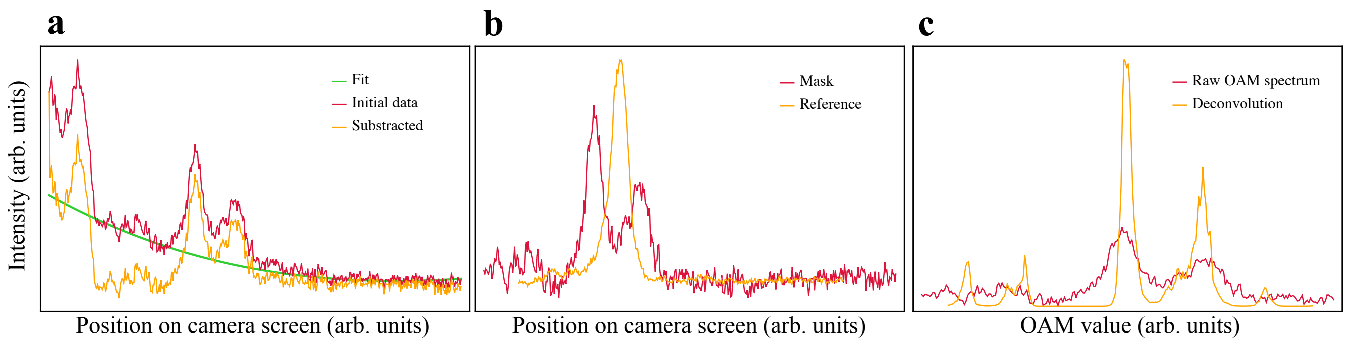

Supplementary Figure 2: **Obtained OAM spectra at various stages of its processing.** **a** Raw data obtained from the sorter's output along with the fitted background and the background subtracted data. **b** Background subtracted data along with the reference zero-OAM electron beam used for the sorter's calibration. **c** Raw calibrated OAM spectrum and its deconvoluted version.

Supplementary Note 1: Experimental Configuration

Here, we provide a more detailed version of our electron sorter. Our implemention was configured within a FEI Titan HOLO TEM electron miscroscope which has the extra lens required to compensate for the beam's diffraction in the plane of the sorter's phase elements. The position of the microscope's lenses along with the phase elements used to perform the mapping and how they influence the electron beam are provided in Supplementary Figure **1**. Let it be noted that the microscope's Cs lens is in a

pass-through configuration. Namely, the electrons exiting the lens are in the same state as when they entered it. That being said, the Cs lens is depicted below as a thin lens with a very long focal length.

The full lens configuration was calculated using the EASYLENS software that is currently under development. The configuration is derived from the microscope's LOW mag configuration and uses an exceptionally large excitation of the Extra lens. The first hologram is placed in the microscope's C2 aperture to produce our test wavefunctions. A second hologram, i.e. the sorter, is placed in the microscope's sample position. In this way the hologram can be easily adjusted in terms of rotations. The third hologram, i.e. the sorter-corrector, is placed in the microscope's SAD (selected area diffraction) aperture. The final OAM spectrum is formed on a CCD camera in image mode where the beam is observed in diffraction mode.

Supplementary Note 2: Sorter and corrector holograms

The required unwrapping process can be achieved by means of a log-polar coordinate transformation through the use of two coupled diffractive holograms. However, due to some differences between the optical sorter and our electron microscope-based apparatus, we must resort to bringing slight adaptive corrections to the phase grating of these holograms. On one hand, the phase corresponding to the first one is modified to

$$\Lambda_1 = \varphi_0 \,\mathrm{sign}\left(\sin\left(2\pi\, a\left[y \arctan\left(\frac{y}{x}\right) + x\ln\left(\frac{\sqrt{x^2+y^2}}{b}\right) + x\right]\right)\right) \tag{1}$$

where a and b are two parameters scaled to optimize the method's experimental efficiency while sign denotes the sign function. On the other hand, the added phase associated with the second hologram becomes

$$\Lambda_2 = \varphi_1 \,\mathrm{sign}\left(\sin\left(2\pi\, a\, b\, \exp\left(-2\pi\frac{u}{a}\right)\cos\left(2\pi\frac{v}{a}\right)\right) + 2\pi\, c\, v\right), \tag{2}$$

where $u = -a\ln\left(\sqrt{x^2+y^2}/b\right)$, $v = a\arctan\left(y/x\right)$, and c consists of an additional scaling parameter. For our sorter, we used parameters of $a = 2$, $b = 0.01$, and $c = 0.6$. The factors φ_0 and φ_1 in the phase expressions of both elements should take values of π in order to maximize their respective diffraction efficiencies. Moreover, in order to adapt this method to a transmission electron microscope (TEM), supplementary sets of lenses and apertures were also added.

Supplementary Note 3: Devices to generate test wavefunctions

To generate OAM carrying electrons, a $\exp\left(i\,\alpha(\varphi)\right)$ term must be added to the formulation of their wavefunctions, where $\alpha(\varphi)$ is a function depending on the azimuthal coordinate ($\varphi \in [0, 2\pi]$). To do so, we employ phase masks which will introduce some losses to the intensity of the electron beam which can be attributed to absorption. We modify the term imparted to the wavefunctions to $\exp\left(i\,\alpha(\varphi) - a(\varphi)\right)$, where a is a positive constant accounting for any absorption related effects.

Two-level masks

The phase designed to be added by a two level mask is defined by

$$\alpha(\varphi) = \begin{cases} 0 & 0 < \mathrm{mod}(n\varphi, 2\pi) < \pi \\ \delta_0 & \pi < \mathrm{mod}(n\varphi, 2\pi) < 2\pi \end{cases}. \tag{3}$$

where $\mathrm{mod}(x, b)$ yields the remainder of the division of x by b and n is an integer. It thus follows that such a plate's influence on an electron's wavefunction is provided by the terms

$$\widetilde{\psi}(\varphi) = \begin{cases} 1 & 0 < \mathrm{mod}(n\varphi, 2\pi) < \pi \\ \exp(-a + i\,\delta_0) & \pi < \mathrm{mod}(n\varphi, 2\pi) < 2\pi \end{cases}. \tag{4}$$

To obtain the OAM components of a beam generated by such a mask, we must find the expansion coefficients of this term's Fourier series expanded in φ, namely

$$c_\ell = \frac{1}{2\pi}\int_0^{2\pi} \widetilde{\psi}(\varphi)\exp(i\,\ell\varphi)\,d\varphi \tag{5}$$

$$= \begin{cases} \frac{n}{\ell\pi}[-1+\exp(-a+i\,\delta_0)] & \ell = nm \\ 0 & \ell \neq nm \\ \frac{1}{2}[1+\exp(-a+i\,\delta_0)] & \ell = 0 \end{cases} \tag{6}$$

where m is an odd integer. For non-absorptive masks, i.e. $a = 0$, these coefficients become

$$c_\ell = \begin{cases} \frac{2n}{\ell\pi}(\exp(i\,\delta_0/2)\sin(\delta_0/2)) & \ell = nm \\ 0 & \ell \neq nm\ . \\ \exp(i\,\delta_0/2)\cos(\delta_0/2) & \ell = 0 \end{cases} \tag{7}$$

Due to their well-defined periodicity, these holograms have very strong selection rules.

Spiral masks

These masks are designed to add a phase of $\alpha(\varphi) = (\delta_0 \mathrm{mod}(n\varphi, 2\pi))/2\pi$ to electron wavefunctions. As in the case of two-level masks, we use the corresponding Fourier coefficients to determine the OAM content acquired by electrons upon propagation through this type of element. These coefficients are provided by

$$|c_\ell| = \begin{cases} \frac{\sin\left(\frac{2\pi\ell}{n}+\delta_0\right)}{\ell+\delta_0/2\pi}, & \ell = nm \\ 0, & \ell \neq nm \end{cases} \tag{8}$$

where m is a positive integer. For $\delta_0 = 2\pi$, the hologram produces a vortex beam carrying OAM of $\ell = n$.

Supplementary Note 4: OAM spectrum processing

Experimental OAM spectra obtained from the sorter were analyzed using conventional spectroscopy techniques. Background noise is first subtracted from the obtained signal by fitting it to a third order polynomial and then subtracting the fit from the raw data as shown in Supplementary Figure **2-a**. The OAM scale of the spectra is then calibrated using data obtained from a reference beam. In our case, this reference consisted of a superposition of ±4 OAM states and a reference beam carrying no OAM, both of which are depicted in Supplementary Figure **2-b**. The calibrated signal is then deconvoluted using the maximum entropy method after clipping negative values in the spectra. The effect of the employed deconvolution algorithm is shown in Supplementary Figure **2-c**. Finally, a binning procedure is conducted by assembling all pixels between values of $\ell - 0.5$ and $\ell + 0.5$ from which we obtain discretized OAM spectra. Optimizations of the binning offsets are also performed in order position the spectra's maxima in the centre of their respective bins.

Supplementary Note 5: Analysis of the interaction between the dipole and electrons

Phase added by a Magnetic Dipole onto an Electron Beam

Here we provide a rough derivation of the term $g(r,\varphi)$ from Eq. (2)) introduced by a magnetic dipole to an electron wavefunction. Consider an electron traveling along the z axis and a magnetic dipole lying in the x, y plane such that its magnetic dipole moment $\mathbf{m}$ is oriented along the x axis, i.e. $\mathbf{m} = \mathcal{M}\,\mathbf{e}_x$, where $\mathbf{e}_x$ is the unit vector along the x axis. The phase acquired by the electron upon propagating through the dipole's magnetic field is given by the path integral $\chi = (e/\hbar)\int_C \mathbf{A}\cdot d\mathbf{x}$, where $\mathbf{A}$ is the dipole's vector potential which is given by $\mathbf{A}(\mathbf{x}) = (\mu_0\,\mathbf{m}\times\mathbf{x})/(4\pi\,|\mathbf{x}|^3)$, where $\mathbf{x} = r\,\mathbf{e}_r + z\,\mathbf{e}_z$ and (r,φ,z) are the cylindrical coordinates. Because the electron travels along the z direction, then χ is to be calculated along the z axis and only the z component of

the vector potential, A_z, becomes relevant to the calculation, i.e. $\chi = (e/\hbar)\int_{-\infty}^{+\infty} A_z\, dz$. Given that $\mathbf{m}\times\mathbf{x} = -\mathcal{M}\, z\, \mathbf{e}_y + \mathcal{M}\, r \sin(\varphi)\, \mathbf{e}_z$, then it follows that our phase is given by

$$\chi = \frac{e}{\hbar}\int_{-\infty}^{+\infty} \frac{\mu_0}{4\pi}\frac{\mathcal{M}\, r \sin(\varphi)}{(r^2+z^2)^{3/2}}\, dz = \frac{e}{\hbar}\frac{\mu_0}{4\pi}\frac{2\mathcal{M}\, r\sin(\varphi)}{r^2} = \frac{e\,\mu_0\,\mathcal{M}}{h\, r}\sin(\varphi) \tag{9}$$

which corresponds to Eq. (2).

Predicting the OAM Expansion Coefficients

We can obtain the expansion coefficient c_ℓ attributed to the phase added by a magnetic dipole onto an electron beam by performing a Fourier transform of the term, g as introduced in Eq. (2), added by the dipole onto the wavefunction of the electrons. This transformation must be performed in terms of the Cartesian coordinates (u, v) of the plane in which the beam is unwrapped. It therefore follows that these coefficients are given by

$$c_{p,\ell} = \int_{-\infty}^{+\infty}\int_{-\infty}^{+\infty} \exp(i\chi(r(u))\sin\varphi(v))\exp(i\,\ell v)\exp(i\,up)\, du\, dv \tag{10}$$

Given that our transformed coordinates are defined by $u = -a\ln(r/b)$ and $v = a\varphi$, we perform according variable substitutions in order to obtain an expression for the coefficients in terms of polar coordinates. For simplicity, we assume that a and b are equal to 1 and obtain

$$c_{p,\ell} = \int_0^{2\pi}\int_0^{\infty} \exp(i\chi(r)\sin\varphi)\exp(i\,\ell\varphi)\exp(-i\,\ln(r)p)\, dr\, d\varphi \tag{11}$$

In the case where an intermediate aperture is added to sorter's configuration, we may model this addition by modulating the above integrand by a Gaussian function or more precisely a radial step-function $W(r)$. In the case where this aperture is sufficiently narrow, we may consider that the main coefficients in the OAM spectrum are for p values satisfying $p \approx 0$ hence allowing us to neglect any other values of p. Based on these considerations, we may further simplify our expansion coefficients to

$$c_\ell \approx c_{0,\ell} = \int_0^{2\pi}\int_0^{\infty} \exp(i\chi(r)\sin\varphi)\exp(i\,\ell\varphi)W(r)\, dr\, d\varphi. \tag{12}$$

We then expand $\exp(i\chi(r)\sin\varphi)$ using the Jacobi-Anger identity. Due to the orthonormality between the exponentials in the resulting expression, we may further simplify the expansion coefficient to

$$c_{0,\ell} = \int_0^{\infty} J_\ell(\chi(r))W(r)\, dr \approx \int_{r_0-\sigma}^{r_0+\sigma} J_\ell(\chi(r))\, dr \tag{13}$$

where the bounds of integration $r_0 - \sigma$ and $r_0 + \sigma$ are used to approximate the influence of the modulating apperture function on the expansion coefficients. From this expression, we observe that $c_{0,\ell} \approx 0$ when $\chi(r) \ll \ell$. Likewise, in the case where $\chi(r) \gg \ell$, the integrand oscillates rapidly and integration over these larger r values will likewise yield negligible contributions. Therefore, these $c_{0,\ell}$ coefficients will only hold significant values over a range where $\chi(r)$ is near $|\ell|$.
